# The Musashi-1–type 2 deiodinase pathway regulates astrocyte proliferation

**DOI:** 10.1016/j.jbc.2024.107477

**Published:** 2024-06-13

**Authors:** Petra Mohácsik, Emese Halmos, Beáta Dorogházi, Yvette Ruska, Gábor Wittmann, Antonio C. Bianco, Csaba Fekete, Balázs Gereben

**Affiliations:** 1 Laboratory of Molecular Cell Metabolism, HUN-REN Institute of Experimental Medicine, Budapest, Hungary; 2 Laboratory of Integrative Neuroendocrinology, HUN-REN Institute of Experimental Medicine, Budapest, Hungary; 3 Section of Adult and Pediatric Endocrinology and Metabolism, University of Chicago, Chicago, Illinois, USA

**Keywords:** thyroid hormone, glial cell, cell proliferation, glioblastoma, deiodinase, central nervous system, protein–mRNA interaction

## Abstract

Thyroid hormone (TH) is a critical regulator of cellular function and cell fate. The circulating TH level is relatively stable, while tissue TH action fluctuates according to cell type–specific mechanisms. Here, we focused on identifying mechanisms that regulate TH action through the type 2 deiodinase (D2) in glial cells. *Dio2* mRNA has an unusually long 3′UTR where we identified multiple putative MSI1 binding sites for Musashi-1 (MSI1), a highly conserved RNA-binding cell cycle regulator. Binding to these sites was confirmed through electrophoretic mobility shift assay. In H4 glioma cells, shRNA-mediated MSI1 knockdown increased endogenous D2 activity, whereas MSI1 overexpression in HEK293T cells decreased D2 expression. This latter effect could be prevented by the deletion of a 3.6 kb region of the 3′UTR of *Dio2* mRNA containing MSI1 binding sites. MSI1 immunoreactivity was observed in 2 mouse *Dio2-*expressing cell types, that is, cortical astrocytes and hypothalamic tanycytes, establishing the anatomical basis for a potential *in vivo* interaction of *Dio2* mRNA and MSl1. Indeed, increased D2 expression was observed in the cortex of mice lacking MSI1 protein. Furthermore, MSI1 knockdown–induced D2 expression slowed down cell proliferation by 56% in primary cultures of mouse cortical astrocytes, establishing the functionality of the MSI1–D2–T3 pathway. In summary, *Dio2* mRNA is a target of MSI1 and the MSI1–D2–T3 pathway is a novel regulatory mechanism of astrocyte proliferation with the potential to regulate the pathogenesis of human glioblastoma.

Despite the relatively stable level of thyroid hormone (TH) in the circulation, a tightly controlled cell type–specific regulatory machinery allows for the fine-tuning of TH action to meet the spatially and timely changing needs of specific cell types (1). This machinery involves membrane transporters, deiodinase enzymes, TH receptors, and their coregulators. The component with the largest dynamic range is the local TH metabolism catalyzed by the deiodinase family of enzymes (2). These mechanisms are especially relevant in the cerebral cortex (CTX) where most T3 is generated locally from T4, which is the main product of the thyroid gland that must be activated to T3 to efficiently bind to the TH receptor and trigger its transcriptional effects (3). In the brain, the T4 to T3 conversion is mediated by the type 2 deiodinase enzyme (D2) expressed exclusively in glial cells, astrocytes, and hypothalamic tanycytes, and this process is under multilevel regulation (1, 4, 5).

TH action is a key regulator of cellular function. In the brain, T3 decreases cyclin-D1 levels and elevates nerve growth factor levels (6, 7), and therefore D2-mediated T3 production has the potential to affect local cell differentiation.

D2 is encoded by an unusually long (∼6–7 kb) *Dio2* mRNA that contains an 800-bp–long coding region upstream of an unusually long 3′UTR (8, 9), which potentially undergoes posttranscriptional regulation. The D2 protein is highly conserved across chicken, rodents, and humans (9). Although sequence homology is much lower between the *Dio2* UTRs, there is a well-documented evolutionary conservation of UTR-driven regulation of D2 expression. This includes the regulation of D2 translation by short ORFs in the 5′UTR and the SECIS element in the 3′UTR along with the role of mRNA instability motifs in the 3′UTR (1, 10).

Musashi-1 (MSI1) is a neural RNA-binding protein that plays an important role in development and could affect TH signaling through D2 regulation. MSI1 expression promotes proliferative pathways in glial cells of the brain and retina photoreceptors, as well as in a variety of other tissues and cells, including the intestine, pancreatic beta cells, and the gastrointestinal epithelium (11, 12, 13, 14). In addition, increased MSI1 expression was associated with the formation and rapid progression of different tumors, particularly glioblastoma (15, 16, 17, 18, 19).

The coexistence of MSI1 and D2 in the adult brain, the long *Dio2* 3′UTR as a potential target of RNA-binding proteins, and their potential opposite roles in the determination of cell fate prompted us to investigate in which settings *Dio2* could be a downstream target of MSI1 with the potential to regulate cell fate.

## Results

### MSI1 regulates D2 expression

**Figure 1:**
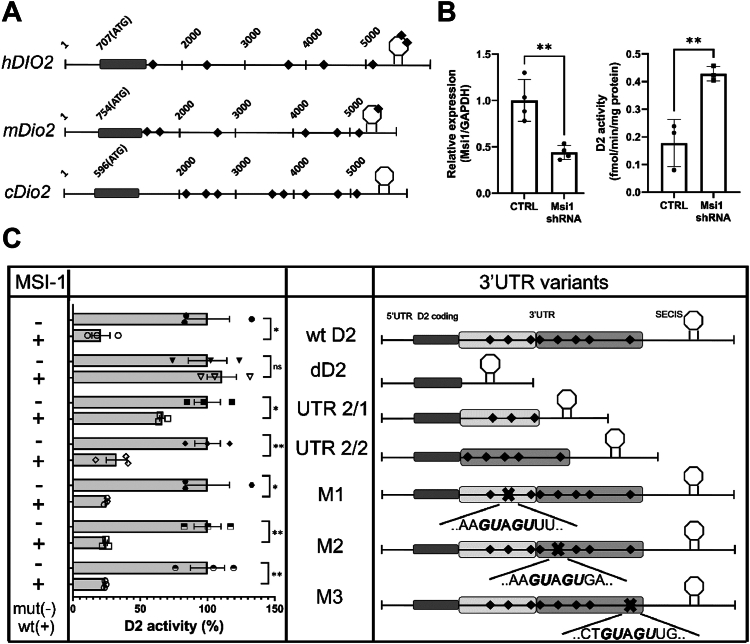
**MSI1 downregulates D2 activity in a 3′UTR-dependent manner.***A*, schematic figure of putative MSI1 binding sites in the 3′UTR of the human (h), mouse (m), and chicken (c) *Dio2* mRNA. The potential MSI1 binding sites are shown as *black diamonds*. *B*, *Msi1* expression decreased and D2 activity increased in H4 glioma cells after knockdown of endogenously expressed *Msi1* with shRNA. Mean ± S.D. ∗∗*p* < 0.01; Student’s *t* test; n = 3. *C*, D2 activity decreased in HEK293T cells after transient coexpression of c*Dio2* 3′UTR variants with WT mouse MSI1 (wt MSI1). As controls, a binding site mutant mouse MSI1 (mut MSI1) was used. Mutations are indicated by X, changes are shown in *bold* and *italics* below the sites, and GUAGU was replaced by CAACA. Positions of M1, M2, and M3 mutations (referring to the first G nucleotide indicated on the Figure) are 2614, 3636, and 4881 nt in the *cDio2* mRNA, respectively. Mut MSI1 coexpressing samples were used as control in case of each single D2 3′UTR variant and compared with Students *t* test. Results are presented in percentage of control. Mean ± S.D. ∗*p* < 0.05; ∗∗*p* < 0.01; ∗∗∗*p* < 0.001 with Students *t* test; n = 3. D2, type 2 deiodinase enzyme; MSI1, Musashi-1.

Sequence analysis identified numerous putative MSI1 binding sites in the mouse, human, and chicken *Dio2* 3′UTR (Fig. 1*A*). To functionally test the impact of MSI1 on D2, H4 neuroglioma cells, endogenously coexpressing MSI1 and D2, were transfected to transiently express MSI1 shRNA. D2 catalytic activity was used to assess the levels of D2 protein expression. Suppression of MSI1 resulted in a 2.4-fold increase in D2 activity (Fig. 1*B*). Next, we tested the effects of MSI1 on *Dio2* mRNA containing different 3′UTR variants. Coexpression of wt MSI1 with a *Dio2* mRNA containing a full-length 3′UTR (wt UTR) decreased D2 expression by 65% compared to negative controls expressing an inactive MSI1 mutant (mut MSI1; harboring mutations in essential positions for RNA binding in both RNA-binding domains (20)). In contrast, MSI1 did not change D2 expression when a 3.6 kb region of the *Dio2* 3′UTR was removed (dD2). MSI1 decreased D2 expression moderately, by 34%, when the first half of the 3′UTR was retained (UTR 2/1), while it exerted a more robust 68% decrease when the second half of the UTR was preserved (UTR 2/2). This suggests that the binding sites located in the UTR 2/2 are more effective in D2 suppression, but multiple MSI1 binding sites in both regions are needed to evoke their full repressive effect on D2 expression (Fig. 1*C*).

Three potential binding sites were further analyzed by targeting the core motif with point mutations. Three single *Dio2* 3′UTR muts were generated and tested in HEK293T cells. None of the mutations had a significant effect on D2 expression and could not restore the baseline enzyme activity when they were coexpressed with wt MSI1 or mut MSI1, suggesting that the binding is promiscuous and deletion of one site can be compensated by binding to others (Fig. 1*C*).

### MSI1 directly binds the Dio2 mRNA

**Figure 2:**
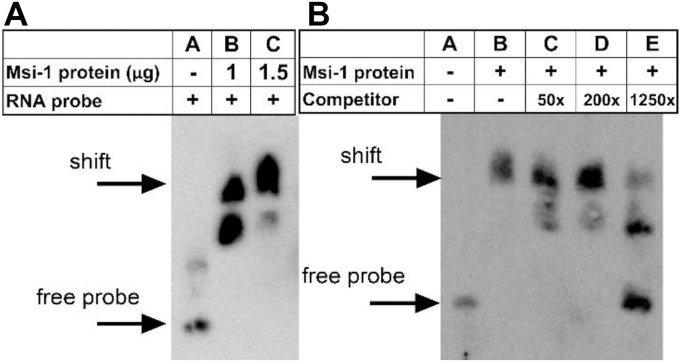
**MSI1 directly binds the 3′UTR of the *Dio2* mRNA. RNA electromobility shift assay.***A*, lane *A*: free probe, 3′biotinylated [Btn] RNA probe GUUUAUAGCUAGAUUAGUUUAGCUGAAAGUUAAGUAGUUUGCAA[Btn] nt:2582-2625 bp of the *cDio2* mRNA. Lane *B*: biotinylated probe+1 μg recombinant mouse MSI1 protein resulted in two shifted bands. Lane *C*: complete shift: biotinylated probe + 1.5 μg recombinant mouse MSI1 protein. *B*, unlabeled competitor RNA probe was added in increasing molar ratio. Lane *A*: free probe. Lane *B*: probe + 1.5 μg protein. Lane *C*: probe + 1.5 μg + 4 pmol (50× excess) unlabeled RNA probe. Lane *D*: probe + 1.5 μg protein + 16 pmol (200× excess) unlabeled RNA probe. Lane *E*: probe + 1.5 μg protein + 100 pmol (1250× excess) unlabeled RNA probe. MSI1, Musashi-1.

To test whether MSI1 can directly bind the putative MSI1 binding sites of the *Dio2* mRNA, we next used RNA electrophoretic mobility shift assay (EMSA). The use of 1 μg recombinant mouse MSI1 protein resulted in an incomplete shift (Fig. 2*A*; Lane B), while 1.5 μg protein was sufficient to achieve a complete shift of 80 fmol free biotinylated RNA probe containing a fragment of the 3′UTR of the *Dio2* mRNA (Fig. 2*A*; Lane C). Increasing molar amounts of nonlabeled RNA probe could restore free probe fraction in 1250× (100 pmol) excess, confirming effective and direct binding of MSI1 to *Dio2* 3′UTR (Fig. 2*B*).

### MSI1 is expressed in D2-expressing cell types

Having established that MSI1 regulates D2 expression in cultured cells through mRNA binding, we next wished to understand whether this interaction could occur *in vivo*. It is known that *Dio2* mRNA is expressed in astrocytes in various brain regions, while in the hypothalamus its expression is confined to tanycytes (21, 22). MSI1 was shown to be present in glial cells in the brain but a more detailed characterization of MSI1 expression was not available (23, 24).

**Figure 3:**
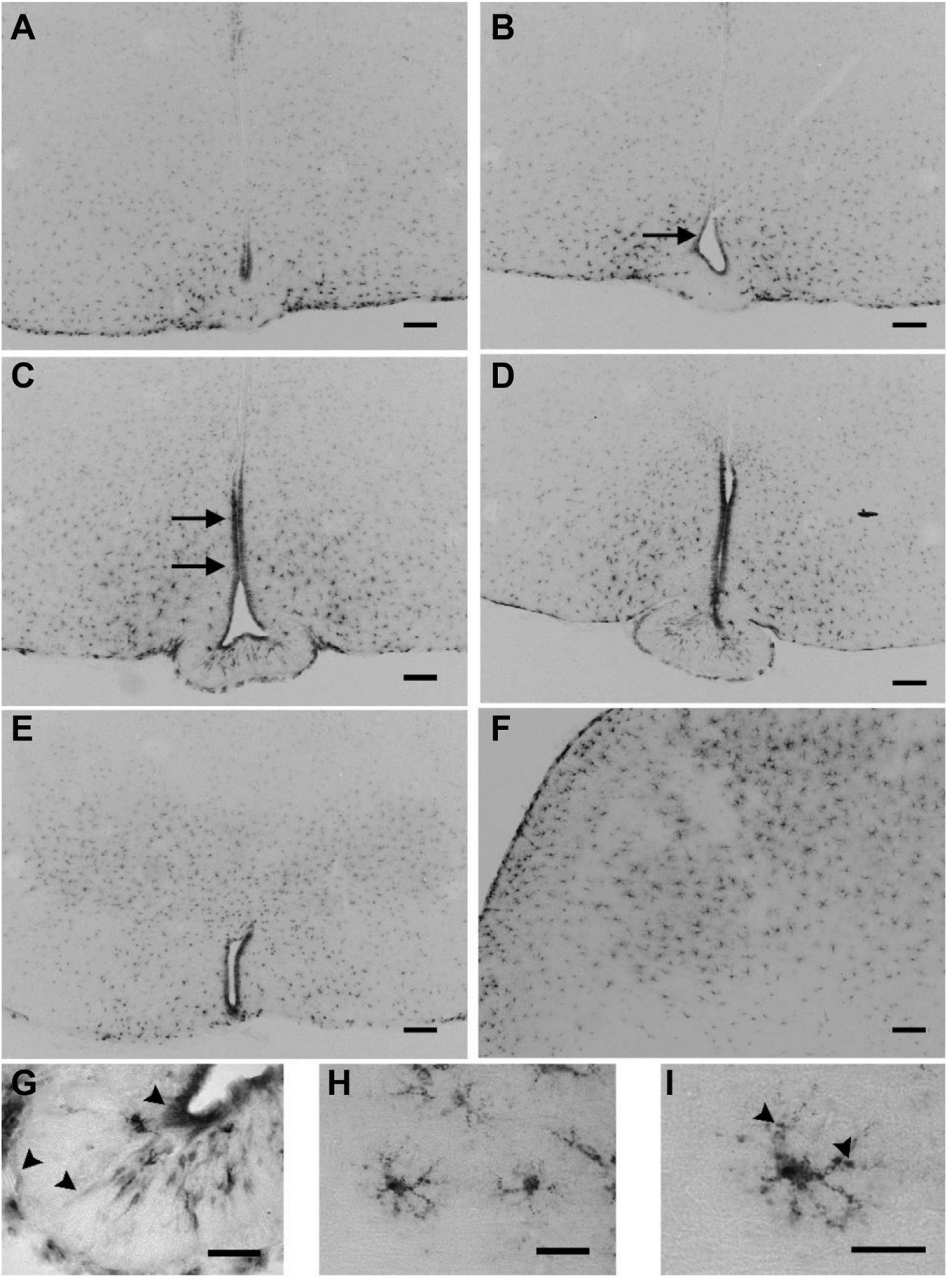
**MSI1 immunohistochemistry in the mediobasal hypothalamus and cortex of C57BL6 WT mice.** MSI1 protein is present in tanycytes at the level of retrochiasmatic area (*A*), and the median eminences (*black arrows*, *B*–*E*). Astrocytes of the mediobasal hypothalamus (*A*–*E*) and cortex (*F*) are also densely labeled. The scale bars on *A*–*F* represent 100 μm. In astrocytes and tanycytes, MSI1 is localized to nucleus, cytoplasm, and it is also present in tanycyte processes, end feets (*black arrowheads*) (*G*), and astrocyte processes with a punctuate pattern (*H* and *I*). *Panel I* shows a magnified region of *H* (the scale bars on *G*–*I* represent 40 μm). MSI1, Musashi-1.

**Figure 4:**
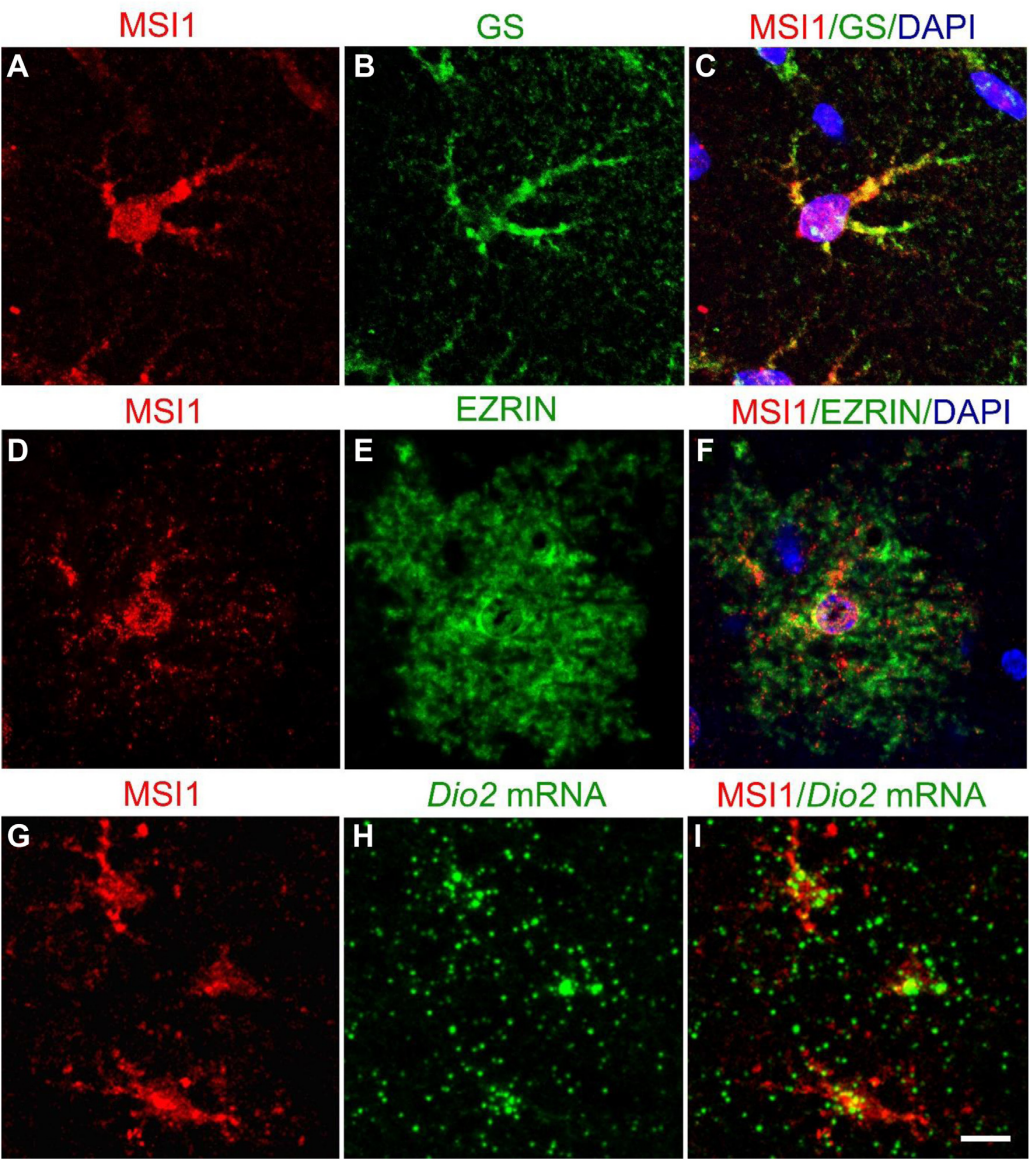
**MSI1 and *Dio2* mRNA are coexpressed in astrocytes of C57BL6 WT mice.** Double-labeling immunofluorescence (IF) detection revealed colocalization of MSI1 and astrocyte markers glutamine synthetase (GS) (*A*–*C*) and ezrin (*D*–*F*) proving that MSI1 is present in mouse cortical astrocytes. FISH of *Dio2* mRNA combined with IF detection of MSI1 protein on 25 μm coronal sections of C57Bl6 mouse brains show colocalization of MSI1 and *Dio2* mRNA in mouse cortical astrocytes. *G*–*I*, the scale bar represents 10 μm (*A*–*I*). MSI1, Musashi-1.

We therefore performed MSI1 immunohistochemistry in mouse brain coronal sections, which revealed strong immunostaining in tanycytes (Fig. 3, *A*–*E*). MSI1 localized mainly to the cell body and nucleus of tanycytes, but it was also abundantly present in β2 tanycyte processes and in end feets closely associated with hypophyseal portal capillaries of the pituitary gland (PIT). We also found widespread MSI1 expression in cortical astrocytes in which nuclei, cell bodies, and processes were also densely stained with a punctuate pattern (Figs. 3, *F*, *H*, and *I* and 4, *A*, *D*, and *G*). In addition, double-labeling immunofluorescent studies documented that MSI1 is expressed in cortical astrocytes, the identity of which was verified through the expression of the glutamine synthetase (GS) (Fig. 4, *A*–*C*) and ezrin (Fig. 4, *D*–*F*). Furthermore, the coexpression of MSI1 protein and *Dio2* mRNA was detected by combined immunocytochemistry and *in situ* hybridization in these cells (Fig. 4, *G*–*I*).

### Brain and PIT-specific disruption of MSI1 expression in Msi1:Msi2 f/f;f/f double-floxed mice

**Figure 5:**
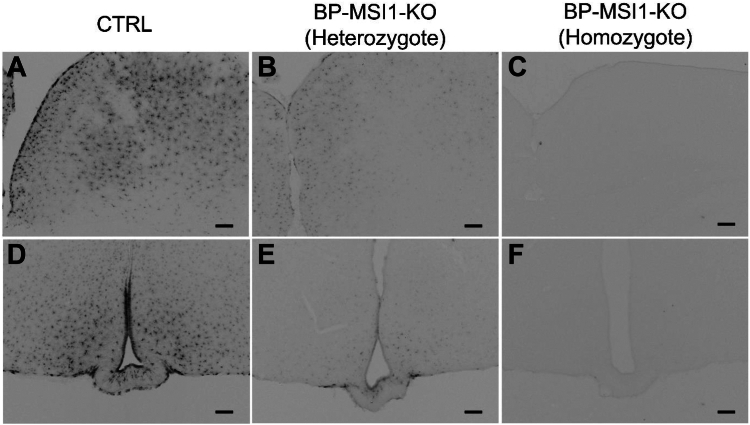
**Ablation of MSI1 in the brain of BP-MSI1-KO mice.** MSI1 immunoreactivity detected by Ni-DAB chromogen on coronal sections of the brain of C57BL6 control (CTRL) (*A* and *D*; source images of *A* and *D* are the reuse of Fig. 3, *C* and *F*, respectively), heterozygote BP-MSI1-KO (*Msi1:Msi2* f/+;f/+) (*B* and *E*), and homozygote BP-MSI1-KO (*Msi1:Msi2* f/f;f/f) (*C* and *F*) mice. Heterozygote BP-MSI1-KO animals have an intermediate level of MSI1 protein in astrocytes and tanycytes (*B* and *E*), while MSI1 is undetectable in the entire brain of homozygote BP-MSI1-KO animals even without crossing them with a Cre-expressing mouse line (*C* and *F*). The scale bar represents 100 μm. BP-MSI1-KO, brain-PIT–specific MSI1 KO; MSI1, Musashi-1; Ni-DAB, nickel-diaminobenzidine.

**Figure 6:**
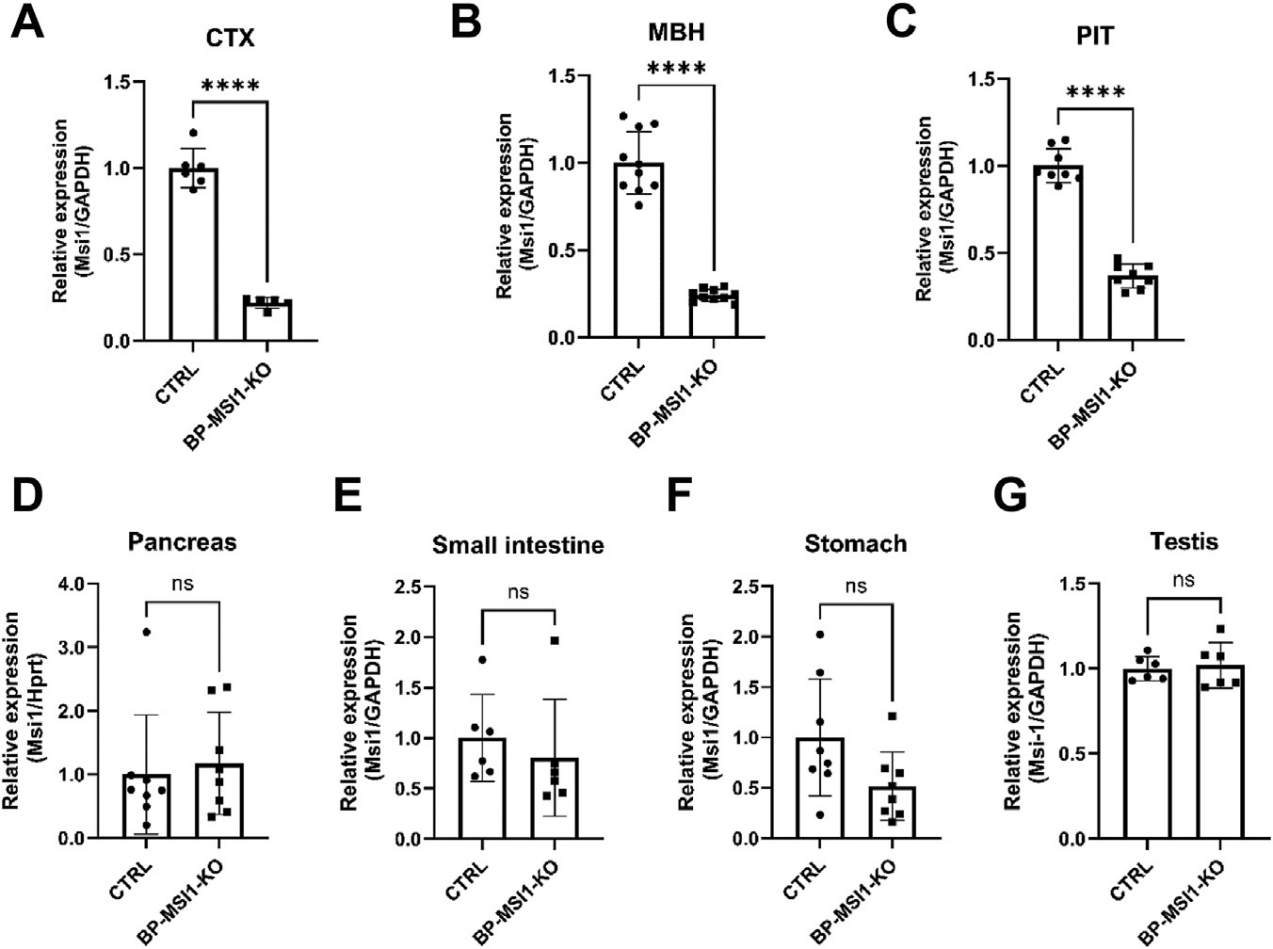
***Msi1* mRNA expression in the brain and peripheral tissues of BP-MSI1-KO mice.***Msi1* mRNA expression level was measured by quantitative polymerase chain reaction in tissues of control (CTRL) and BP-MSI1-KO mice (*Msi1:Msi2* f/f;f/f). *A* and *B*, *Msi1* mRNA level decreased by 80% in the mediobasal hypothalamus (MBH) and cortex (CTX) (n = 6; ∗∗∗∗*p* < 0.0001 and n = 10; ∗∗∗∗*p* < 0.0001 by Student’s *t* test, respectively) in the BP-MSI1-KO mice. *C*, the *Msi1* mRNA expression also decreased in the pituitary of the transgenic mice by 60% (∗∗∗∗*p* < 0.0001; n = 8; by Student’s *t* test). *D*–*G*, the *Msi1* mRNA expression was not significantly affected in the tested peripheral tissues. Mean ± SD. n ≥ 6; by Student’s *t* test. BP-MSI1-KO, brain-PIT–specific MSI1 KO; MSI1, Musashi-1.

To investigate the role of MSI1 in D2 regulation in the mouse brain *in vivo*, we obtained the *Msi1:Msi2* f/f;f/f double-floxed mice (25, 26). We observed that MSI1 protein is undetectable in the whole brain of *Msi1:Msi2* f/f;f/f homozygote animals, and an intermediate, weak expression was present in *Msi1:Msi2* f/+;f/+ heterozygote brains (Fig. 5) even without crossing these mice with any Cre-expressing mouse line. *Msi1* mRNA expression measured with real-time qPCR showed a 5-fold lower *Msi1* levels in mediobasal hypothalamus (MBH) and CTX and 3-fold lower levels in PIT of double-floxed mice than C57Bl6 control animals (Fig. 6, *A*–*C*). In contrast to the dramatic decrease in brain *Msi1*, the expression was not significantly affected in peripheral tissues (Fig. 6, *D*–*G*). Expression of Musashi-2 (*Msi2*), the other member of the Musashi family remained unaffected in the cortex and PIT (Fig. S1).

**Figure 7:**
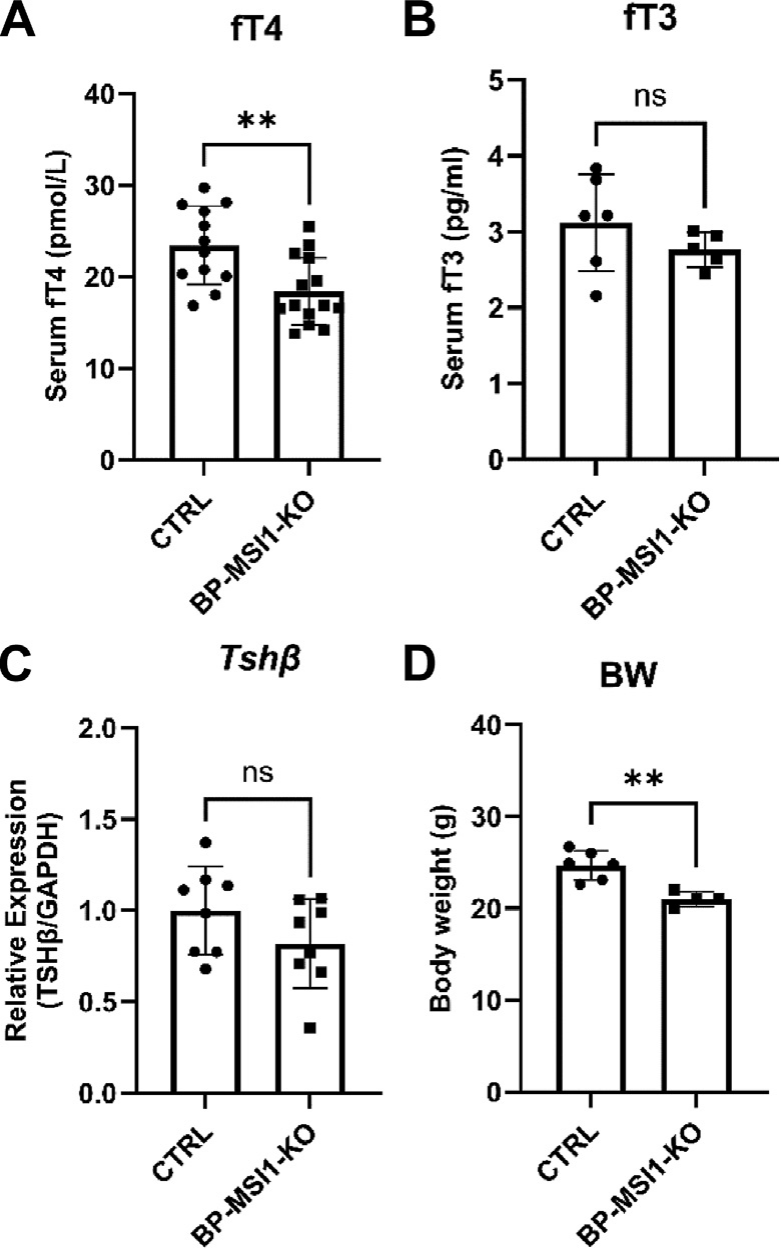
**Characterization of the thyroid hormone status of BP-MSI1-KO mice.***A*, serum fT4 level was slightly decreased in BP-MSI1-KO mice (*Msi1:Msi2* f/f;f/f) (*p* < 0.01; n ≥ 12 by Student’s *t* test). *B* and *C*, serum fT3 level and pituitary *Tshβ* mRNA expression remained unchanged (n ≥ 5). *D*, body weight was slightly decreased in KO animals. Mean ± SD. ∗∗*p* < 0.01; n ≥ 5; by Student’s *t* test. BP-MSI1-KO, brain-PIT–specific MSI1 KO; MSI1, Musashi-1.

Analysis of the *Msi1* promoter of the *Msi1:Msi2* f/f;f/f mice revealed that the short AT-rich 5′-LoxP sequence was inserted into a highly conserved GC-rich region of the promoter (−351 nt to ATG), suggesting that the insertion of the LoxP site destroyed a brain and PIT-specific enhancer region resulting in a brain-PIT–specific KO animal, without affecting MSI1 expression in peripheral tissues. In light of these findings, *Msi1:Msi2* f/f;f/f homozygote mice were used in further experiments as brain-PIT–specific MSI1 KO (BP-MSI1-KO) animals. In these animals, body weight was slightly reduced compared to controls (Fig. 6*D*), whereas fT3 serum levels and *Tshβ* mRNA remained unchanged (Fig. 7, *B* and *C*); serum fT4 levels were only slightly decreased (Fig. 6*A*). Altogether, these data indicate that no significant changes in the global TH economy were observed.

### Role of MSI1 in the regulation of Dio2 mRNA levels and D2 expression in the mouse brain and PIT

**Figure 8:**
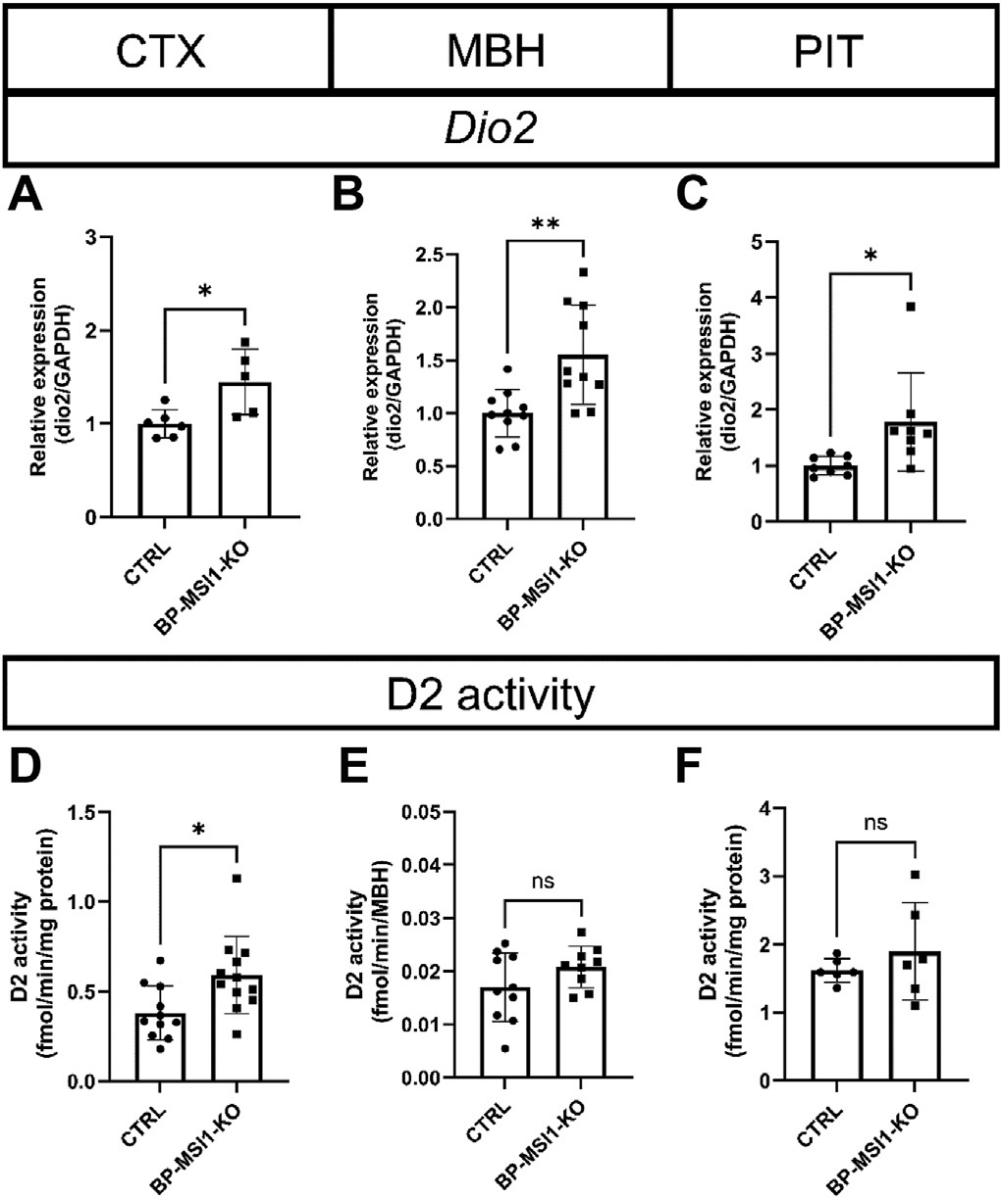
***Dio2* mRNA expression and D2 activity in the brain and pituitary of BP-MSI1-KO mice.***A*–*C*, the expression of *Dio2* mRNA was increased in the cortex (CTX), the mediobasal hypothalamus (MBH) and pituitary (PIT) of BP-MSI1-KO (*Msi1:Msi2* f/f;f/f), and C57Bl6 control (CTRL) mice. *D*, D2 activity was increased in the CTX of BP-MSI1-KO mice. *E*, D2 activity remained unchanged in the MBH and (*F*) in the PIT. Mean ± SD. ∗*p* < 0.5, ∗∗*p* < 0.01, n ≥ 5, Student’s *t* test. BP-MSI1-KO, brain-PIT–specific MSI1 KO; D2, type 2 deiodinase enzyme; MSI1, Musashi-1.

To study the role of MSI1 in the regulation of the D2 enzyme *in vivo*, we studied *Dio2* mRNA levels and D2 expression in the CTX, MBH, and PIT of the BP-MSI1-KO mice. Reduction of MSI1 increased *Dio2* mRNA levels (∼1.5-fold) in all three regions (Fig. 8, *A*–*C*). Notably, the observed change translated into a significantly increased D2 expression in the CTX (Fig. 8*D*), while D2 expression remained unchanged in the MBH (where D2 is exclusively present in tanycytes) and in the PIT (Fig. 8, *E* and *F*).

### MSI1 expression is regulated by the TH

**Figure 9:**
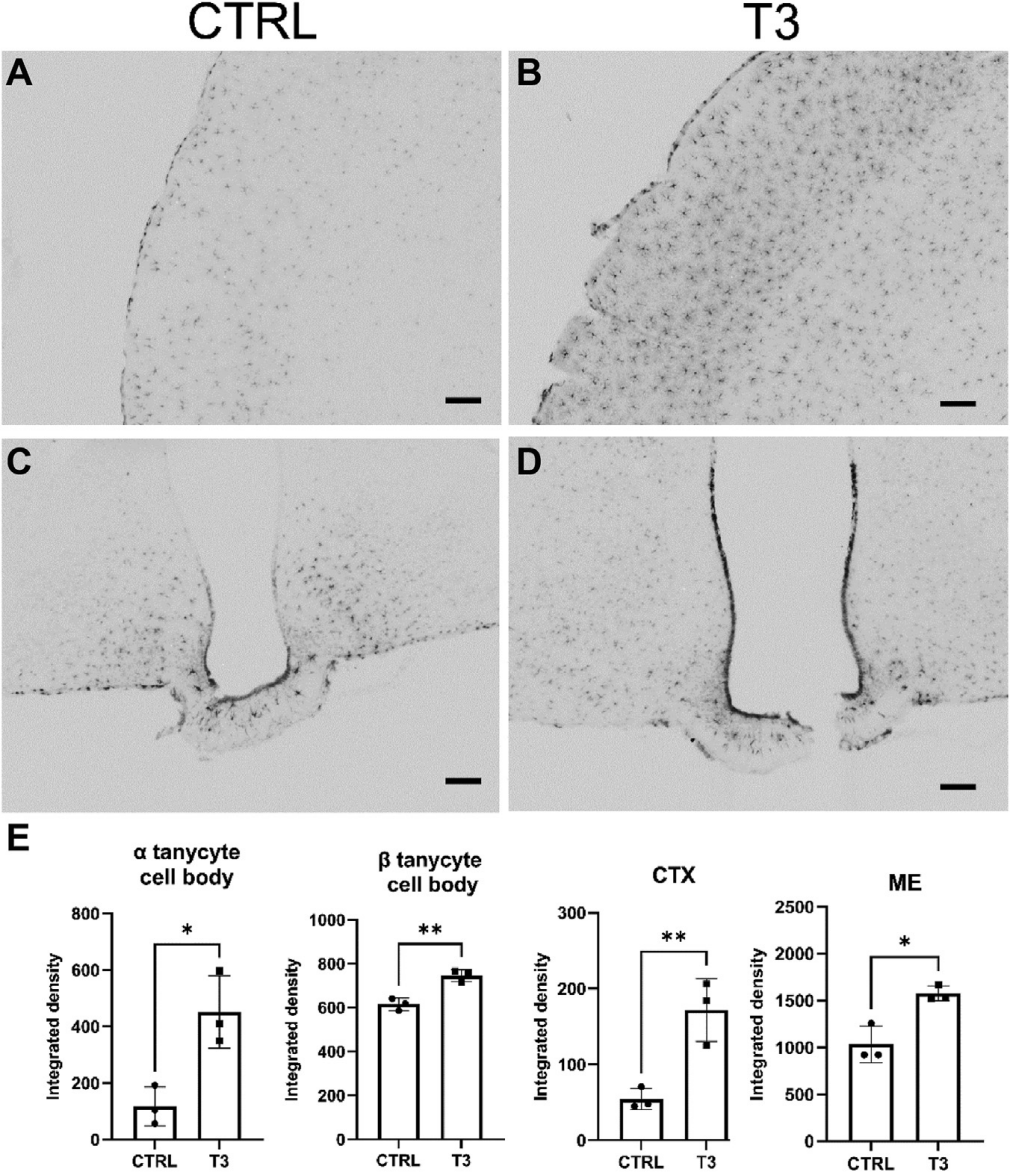
**T3 upregulates MSI1 level in glial cells *in vivo in* C57BL6 WT mice.** One day (24 h) after a single dose of T3 (1 μg/bwg, i.p.), MSI1 immunoreactivity was increased in cortical astrocytes (*A* and *B*) and in all tanycyte subtypes (*C* and *D*) compared to vehiculum-treated C57Bl6 (CTRL) mice detected with Ni-DAB chromogen on coronal sections. The scale bars represent 100 μm. *E*, integrated densities were determined in cell bodies of α and β tanycytes, the median eminence (ME) region, and cortex (CTX). Mean ± SD. ∗*p* < 0.05, ∗∗*p* < 0.01, n = 3, Student’s *t* test. MSI1, Musashi-1; Ni-DAB, nickel-diaminobenzidine.

To test whether generated TH could exert a feedback on MSI1 expression in the brain, mice received a single *i.p*. administration of T3 and later MSI1 protein levels were measured using immunohistochemistry. In cortical astrocytes, MSI1 immunoreaction signal was increased by 3-fold in response to T3 treatment (Fig. 9, *A*, *B*, and *E*). In the hypothalamus, T3 administration resulted in a ∼1.5-fold increase of the integrated density values of MSI1 immunoreactivity in the median eminence (Fig. 9, *C*–*E*). MSI1 level was increased in the cell bodies of tanycytes, by 4-fold in α and 1.2-fold in β tanycytes. In control animals, MSI1 level was higher in β than α tanycytes (Fig. 9*E*).

### The MSI1–D2 pathway regulates astrocyte proliferation

**Figure 10:**
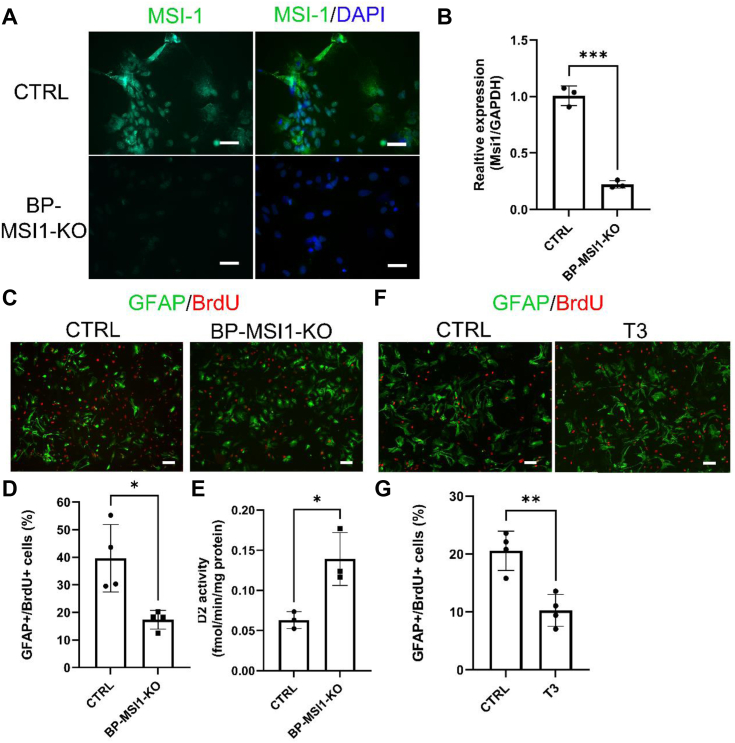
**The MSI1–D2–T3 pathway regulates the proliferation of primary cortical astrocytes.***A*, MSI1/4′,6-diamidino-2-phenylindole immunofluorescence on primary cortical astrocytes of BP-MSI1-KO (*Msi1:Msi2* f/f;f/f) and C57Bl6 control (CTRL) mice. The scale bar represents 40 μm. *B*, *Msi1* mRNA expression in primary astrocytes of BP-MSI1-KO and C57Bl6 control mice. Mean ± SD. n = 3; ∗∗∗*p* < 0.001, Student’s *t* test. *C*, representative images of GFAP/BrdU immunofluorescence on primary cortical astrocytes of BP-MSI1-KO and C57Bl6 control mice. *D*, proliferation rate (given as % of BrdU-labeled GFAP-positive astrocytes) is decreased and (*E*) D2 activity is increased in BP-MSI1-KO astrocytes. Mean ± SD. n ≥ 3; ∗*p* < 0.05; Student’s *t* test. *F*, representative images of GFAP/BrdU immunofluorescence of vehicle and 100 nM T3-treated primary cortical astrocytes of C57Bl6 control mice. *G*, proliferation rate (given as % of BrdU-labeled GFAP-positive astrocytes) was decreased in primary astrocytes of C57Bl6 WT mice treated with 100 nM T3 *versus* vehicle. Mean ± SD. n = 4; ∗∗*p* < 0.01; Student’s *t* test. The scale bar represents 100 μm (*C* and *G*). BP-MSI1-KO, brain-PIT–specific MSI1 KO; BrdU, bromodeoxyuridine; D2, type 2 deiodinase enzyme; GFAP, glial fibrillary acidic protein; MSI1, Musashi-1.

Having established that MSI1 regulates D2 expression in cortical astrocytes and the generated T3 in turn also regulates MSI1 expression, we next investigated whether this MSI1–D2 pathway could effectively modulate TH signaling in target cells. With that in mind, we used bromodeoxyuridine (BrdU) labeling to measure the rate of proliferation of primary cortical astrocytes isolated from BP-MSI1-KO mice. In these cells, MSI1 KO was verified by immunohistochemistry and gene expression analysis (Fig. 10, *A* and *B*). MSI1 KO decreased the proliferation rate of glial fibrillary acidic protein (GFAP)-positive astrocytes, which was accompanied by increased D2 expression (Fig. 10, *C*–*E*). Remarkably, this phenotype was rescued by treatment with T3 (Fig. 10, *F* and *G*).

## Discussion

**Figure 11:**
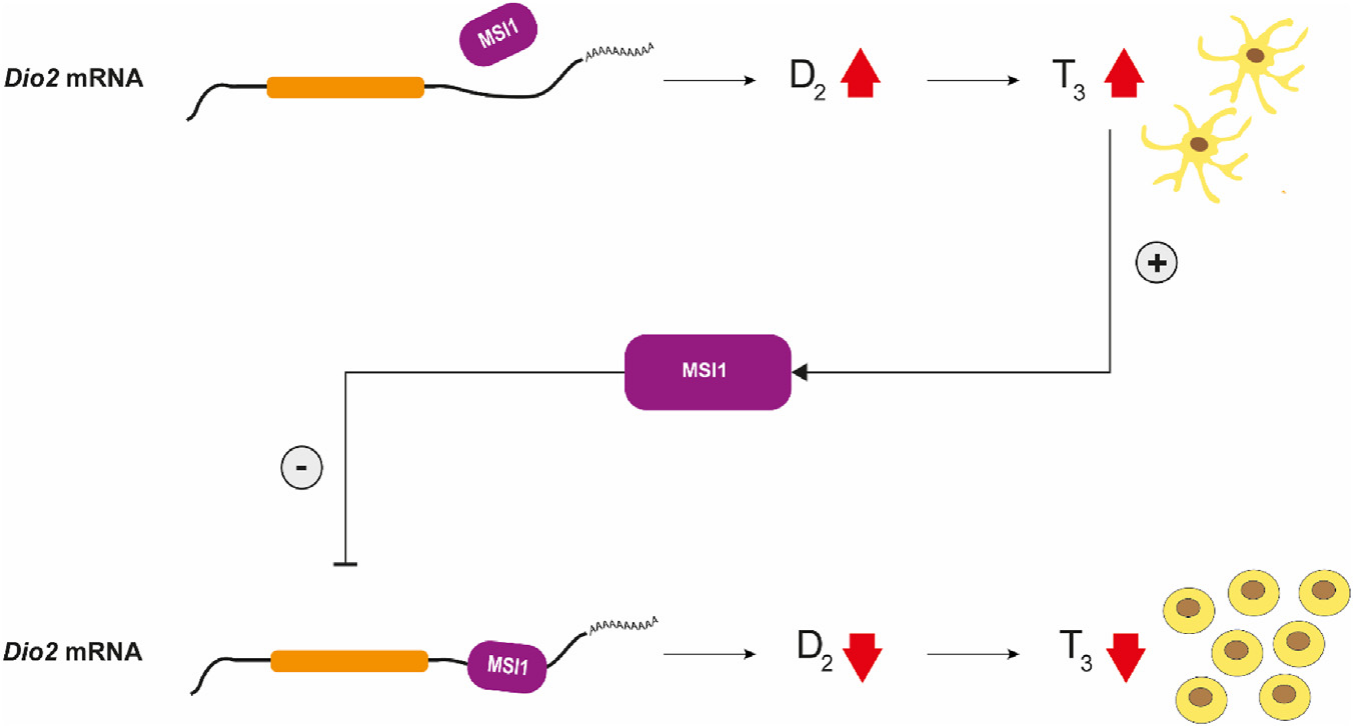
**Schematic depiction of the MSI1–D2–T3 pathway.** MSI1 binding to the 3′UTR of the *Dio2* mRNA decreases D2-mediated T3 generation in cortical astrocytes and promotes cell proliferation. The generated T3 increases MSI1 level and exerts this way a negative feedback on D2-mediated T3 generation. D2, type 2 deiodinase enzyme; MSI1, Musashi-1.

The present data revealed the existence of a MSI1–D2 pathway that regulates glial T3 production and consequently the proliferation of cortical astrocytes (Fig. 11). MSI1 is an RNA-binding protein with two RNA-bindings domains (27, 28) that exerts its effect by direct interaction with the 3′ UTR of target mRNAs, including the *Dio2* mRNA. As in previous studies (29), we observed that MSI1 immunoreactivity is primarily restricted to glial cells, that is, astrocytes throughout the mouse brain and tanycytes in the hypothalamus. Here, we also provide direct evidence that cortical astrocytes coexpress MSI1 and *Dio2* mRNA. In addition, MSI1 immunoreactivity was also detected in a neuronal group in the lateral hypothalamus. MSI1 is localized not only in the cytoplasm and cell nucleus but also distributed along the glial processes with a punctuate pattern. Based on the expression of MSI1 in D2-expressing glial cell types and the presence of numerous putative MSI1 binding sites in the 3′ UTR of the *Dio2* mRNA, we hypothesized that MSI1 regulates D2 expression. Our *in vitro* experiments supported this hypothesis as we observed that changes in MSI1 levels resulted in reciprocal regulation of D2 expression in a 3′ UTR-dependent manner. Furthermore, the direct binding of MSI1 to *Dio2* mRNA 3′ UTR was verified by RNA EMSA.

To study whether MSI1 can regulate D2 expression *in vivo*, we used the BP-MSI1-KO model. This mouse line was generated by floxing *Msi1* and *Msi2* to allow a Cre-dependent gene inactivation (25, 26). Unexpectedly, the immunohistochemical validation of homozygote-floxed animals (without crossing it with a Cre-recombinase expressing driver line) revealed the absence of MSI1 protein in the brain compared to WT mice, while *Msi2* expression remained unaffected. Further screening of peripheral tissues of homozygote animals revealed a normal expression of both MSI1 and MSI2 that explained why previous studies using this model in the retina and intestinal epithelium did not detect this critical off-target phenotype (14, 25). Our analysis suggests that the AT-rich 5′ loxP sequence of the targeting construct inserted (−359 bp) into the highly conserved and ∼90% GC-rich promoter region of MSI1 could be responsible for this phenomenon. This insertion exerted a deleterious effect on promoter activity specifically in the brain and PIT without Cre-mediated deletion, and the floxed animals behaved as a BP-MSI1-KO model. This phenomenon is not unprecedented, for example, transgenic modification of independent sets of enhancers resulted in cell type–specific proopiomelanocortin KO mice (30).

In parallel to our *in vitro* findings, the ablation of MSI1 in the brain and PIT increased *Dio2* mRNA levels in all studied brain areas and also in the PIT. Importantly, however, MSI1 KO exerted a different impact on D2 protein level in hypothalamic tanycytes and cortical astrocytes, since it increased D2 expression in cortical astrocytes while not in hypothalamic tanycytes and in the PIT of BP-MSI1-KO animals. MSI can promote or inhibit translation *via* competing with eukaryotic translation initiation factor 4G or interacting with the poly-A–binding protein and this phenomenon can be accompanied or not both by changes in mRNA level encoding the target protein (20, 31, 32, 33). Our data suggest that the impact of MSI1 on *Dio2* mRNA stability and protein translation could have also cell type–specific aspects and a deeper mechanistic understanding of the observed D2 expression increase in the cortex of BP-MSI1-KO would require further studies.

D2 expression is often distinctly regulated in tanycytes and in extrahypothalamic astrocytes (34). In astrocytes, D2 undergoes homeostatic regulation to ensure local tissue euthyroidism in response to changing peripheral TH levels. In contrast, D2-mediated T3 generation in hypothalamic tanycytes is not homeostatic, its role is to provide an adequate amount of T3 for the regulation of the hypothalamo-pituitary-thyroid axis under various challenges and transmit peripheral signals to hypothalamic neurons and regulate their activity (35, 36). Thus, the different regulation of D2 by MSI1 in the two D2-expressing cell types of the brain could also mirror this dichotomy.

We also studied in the mouse brain, whether T3, the end product of the MSI–D2–T3 pathway, exerted a feedback regulation on this pathway. Our data demonstrated that MSI1 is upregulated by T3 in the mouse cortex and hypothalamus. T3-dependent upregulation of MSI1 was also shown in the rat brain and the amphibian gastrointestinal tract, thus it seems to be a general feature of MSI1 regulation (37, 38). Our data suggests the existence of a novel aspect of the feedback mechanism in astrocytes allowing elevated T3 to downregulate D2 expression based on a posttranscriptional, MSI1-dependent mechanism.

TH is a well-known regulator of cell fate by exerting an impact on cellular differentiation/proliferation in various tissues (39, 40, 41, 42). We showed earlier, that D2-generated T3 regulates cell proliferation in the tibial growth plate, where the proliferation promoting morphogenic protein sonic hedgehog induces WSB-1, an E3 ligase subunit that *via* a posttranslational mechanism increases D2 ubiquitination and consequently decreased D2 activity and T3 availability (41, 43). Since MSI1 is expressed in D2-expressing glial cells, we aimed to learn whether MSI1–D2–T3 pathway could impact astrocyte proliferation. Primary astrocytes isolated from the cortex of BP-MSI1-KO mice showed a decreased proliferation rate compared to astrocytes of control animals which was accompanied by increased D2 expression. Importantly, this could be reversed by T3, indicating that cell proliferation is regulated by an antagonistic functional interaction between MSI1 and D2. This observation suggests a mechanism for previous observations reporting decreased proliferation in MSI KO glioma stem cells and increased proliferation in MSI1-overexpressing murine astrocytes (44).

Mature astrocytes are not dividing in the adult brain; however, there are specific circumstances when glial cells resume stem cell properties and the ability to proliferate. One condition is reactive gliosis after focal ischemia (45) or after traumatic brain injury (46). Astrocytomas and glioblastomas are intensely proliferating and invasive malignant tumor types of the brain that originate from glial cells. Reactive astrocytes and the highly proliferating cells of glia-derived tumors show similar expression patterns to neuronal stem cells and progenitors and most of them highly express MSI1 (44, 45, 47, 48, 49). TH affects glioma cell growth but results about tumor growth and patient survival are inconsistent (50). Our data suggests that the MSI1–D2–T3 pathway regulates TH signaling in the brain but the potential to affect glioma proliferation requires further studies. Targeting the MSI1–D2–T3 pathway may serve as a novel approach to target glioma cells and improve radiotherapy and decrease chemo resistance (51).

## Experimental procdures

### Animals

Seventy-day-old male C57BL6 control and BP-Msi1-KO mice (Msi1:Msi2 f/f;f/f, kindly provided by Dr CJ Lengner) were housed under standard environmental conditions (light between 06:00 h and 18:00 h, temperature 22 ± 1 °C, mouse chow, and water *ad libitum*). C57BL6 mice were *i.p*. injected with a single dose of 1 μg/bwg T3 in vehicle (40 mM NaOH in 0.9% NaCl) or with vehicle alone. Experimental protocols were reviewed and approved by the Animal Welfare Committee at the HUN-REN Institute of Experimental Medicine (PE/EA/1490-7/2017, MÁB-1/2021).

### Immunohistochemistry

Sixty- to seventy-day-old control (C57BL6), homozygote *Msi1:Msi2* f/f;f/f, and heterozygote *Msi1:Msi2* f/+;f/+ male mice were anesthetized with ketamine-xylazine mixture and perfused transcardially with 4% paraformaldehyde (PFA) in 0.1 M PBS, pH 7.4. Brains and sections were handeled and pretreated for immunohistochemistry as previously described (52). Coronal, 25-μm thin sections were immunolabeled for MSI1 using 1:100.000 dilution of rabbit anti-MSI1 primary antibody (Abcam: ab52865, for validation see (53) and the lack of staining in the BP-MSI1-KO brain where *Msi1* mRNA is ablated (Figs. 5 and 6) in 2% normal horse serum (NHS) overnight, at room temperature (RT), followed by incubation with biotinylated donkey anti-rabbit IgG (Jackson, 1:500) followed by incubation in avidin/biotin horseradish peroxidase conjugate (Vectastain, 1:1000) diluted in Tris buffer, pH 7.6 and visualized with nickel-diaminobenzidine development method as previously described (52).

Astrocyte-specific MSI1 expression was detected with double-labeling immunofluorescence (IF). 25 μM coronal sections of 4% PFA fixed brains of 70-day-old C57BL6 control animals were double-labeled with rabbit anti-MSI1 primary antibody (Abcam ab52865; 1:50.000) in combination either with sheep anti-ezrin primary antibody (Bio-Techne AF7239; 1:1000, for validation see (54)) or with guinea pig anti-GS serum (Synaptic Systems 367005; 1:1000, for validation see (55)). MSI1 was visualized with A555-conjugated donkey anti-rabbit IgG (1:500, Invitrogen), while ezrin and GS with A488-conjugated donkey anti-sheep and anti-guinea pig IgGs (1:250, Invitrogen), respectively. Sections were handled as previously described (52).

Brain coronal sections of control (vehiculum-injected) and T3-injected 70-day-old male (C57BL6) mice were prepared and immunolabeled for MSI1 using nickel-diaminobenzidine chromogen as described above. Integrated density of MSI1 immunoreaction signal was measured with Image J software (NIH, https://imagej.net/software/imagej/) in the cell bodies of α and β tanycytes, in the median eminence and cortical areas of similar size.

Four percent PFA-fixed primary astrocytes derived from BP-MSI1-KO and control mice were stained for MSI1 with 1:20.000 dilution of rabbit anti-MSI1 serum (Abcam, ab52865) in 2% NHS overnight at RT, immunoreaction was visualized with incubation in A488-conjugated donkey anti-rabbit IgG (1:250, Invitrogen) for 2 h in 2% NHS at RT. To stain the cell nuclei, the sections were coverslipped with 4′,6-diamidino-2-phenylindole-containing Vectashield mounting medium (Vector Laboratories) after mounting the sections on glass slides.

### Combined *in situ* hybridization and immunohistochemistry

To detect MSI1 protein and *Dio2* mRNA, we applied a previously published protocol (56) for combined IF/FISH with modifications. Briefly, adult (15-week-old) male C57Bl/6J mice (n = 3) were perfused transcardially with 4% PFA. The brains were removed, postfixed in 4% PFA for 4 h, cryoprotected overnight in 20% sucrose in PBS, and then snap-frozen on dry ice. Serial 20-μm coronal sections were cut on a Leica CM3050 S cryostat (Leica Microsystems), collected in cryoprotective solution, and stored at −20 °C until use. Since the FISH conditions were detrimental to the MSI1 immunostaining, we first detected MSI1 with IF, and then proceeded with FISH to detect *Dio2* mRNA. The free-floating sections were treated with 0.5% Triton X-100 and 0.5% H_2_O_2_ for 15 min, rinsed in PBS, and blocked with 2% bovine serum albumine (BSA) in PBS for 20 min. The sections were incubated overnight in the rabbit anti-MSI1 serum (Abcam Ab52865; 1:40.000 in 2% BSA in PBS), rinsed in PBS, and then incubated in Cy3-conjugated donkey anti-rabbit IgG (Jackson, 1:200) for 4 h. The sections were mounted onto Superfrost Plus glass slides and desiccated overnight at 42 °C before FISH. Sections were hybridized for *Dio2* mRNA with the cocktail of two nonoverlapping digoxigenin-labeled antisense riboprobes, the first probe corresponding to 1504 bp to3521 bp and the second to 3519 bp to 5346 bp of the mouse *Dio2* mRNA (NM_010050.4). The DNA templates for the probes were generated by Taq polymerase reaction on mouse cortex complementery DNA (cDNA) and subcloned to pGemT vector. The hybridization procedure followed the published protocol (56) except that 2 μg/ml proteinase K was used in the prehybridization and 2 μg/ml RNase A in the posthybridization phase. The digoxigenin-labeled probes were detected with Fab fragments of peroxidase-conjugated sheep anti-digoxigenin antibody (Merck; 1:100) and subsequent biotinylated tyramide amplification described previously (57), followed by incubation in Alexa 488-Streptavidin (1:500, Thermo Fisher Scientific). Sections were coverslipped with SlowFade Diamond mountant (Thermo Fisher Scientific). Although the Cy3-fluorescence faded noticeably during the FISH procedure, sufficient signal remained to detect MSI1 in cortical astrocytes. Sections were examined with confocal microscopy.

### Polymerase chain reaction

Real-time quantitative polymerase chain reaction (qPCR) was perfomed as previously described (36). In short, RNA was isolated with Nucleospin RNA isolation kit (Macherey-Nagel) from brain and peripheral organ samples and from primary astrocytes according to kit protocol. One microgram of RNA (100 ng of MBH RNA) was reverse transcribed with high capacity cDNA reverse transcription kit (Applied Biosystems), and cDNA concentration was determined with Qubit ssDNA assay kit (Invitrogen) using Qubit fluorimeter. qPCR reactions containing 10 ng cDNA, 10 μl 2× Taqman Universal PCR Master Mix (Applied Biosystems), and 1 μl gene-specific probe (Table S1) in 20 μl final volume were amplified in ViiA 7 real-time PCR System (Applied Biosystems) instrument and results were analyzed with comparative Ct method using *Gapdh* and *Hprt* as housekeeping gene that were stable with low variability under the study conditions. PCR efficiency of premade Taqman assays is 1 by design.

Exact location of 5′loxP site within the promoter region of MSI1 was mapped by sequencing Taq PCR product (forward primer: CGGACTGGGAGAGGTTTCTT reverse primer: ACCAGGAATCAGGGGAGCT) produced on genomic DNA extracted from Msi1:Msi2 f/f;f/f mouse tail.

### Cell lines and transfection

H4 human neuroglioma cells (provided by M. LaVoie, Brigham and Women’s Hospital (58)) were seeded in 6-well plates using 2∗10^5^ cells/well the day before transient transfection. For one transfection reaction 8 μl Extreme Gene HF transfection reagent (Roche), 3000 ng total DNA (500 ng pGIPZ or shRNA [Open Biosystems, clone ID: V3LHS_642990] and 2500 ng carrier DNA) was used. Puromycin (1.5 μg/ml)-containing selection media was applied 48 h posttransfection, and cells were grown for 4 days. Cell pellets were collected and stored at −80 °C until D2 activity measurement and qPCR analysis.

HEK293T cells (provided by PR Larsen, Brigham and Women’s Hospital (43) were seeded using 4∗10^5^ cells/well 1 day before transfection in complete growth medium in 6-well plate. Cells were transfected with Extreme gene HF transfection reagent (Roche) using 3000 ng total DNA (30 ng cD2 3′ UTR variant construct, 10 ng D15 plasmid, 10 ng SEAP, 1200 ng wtMsi1/mutMsi1, 1750 ng carrier DNA/well). Forty eight hours after transfection, culture medium was collected for SEAP enzyme activity measurement. The cells were collected and pellets were stored at −80 °C until D2 enzyme activity measurement. SEAP enzyme activity was measured with NovaBright Phospha-Light EXP assay kit (Invitrogen) from 20 μl of cell culture supernatant according to manufacturer’s instructions using Luminoscan Ascent Luminometer (Thermo Fisher Scientific). D2 activity data were normalized to this transfection control.

### Expression constructs

UTR studies: Plasmid DNAs expressing WT (wt MSI1, PCDNA3-FLAG-MSI1) and double binding site mut (mut MSI1, where both RNA-binding domains were inactivated by a PheA for Leu change in amino acid positions 63, 65, 68, 152, 154, and 157; PCDNA3-FLAG-MSI1-mutR1R2) mouse MSI1 protein (20) were kind gift of Dr H. Okano. WT chicken *Dio2* mRNA (33–6094 nt) in D10 vector was modified to prepare UTR variants. The ΔD2 (dD2) variant was produced by removing a 3.6 kb UTR fragment between the end of the coding region and SECIS element (10). First (D2 2/1; 1466–3332 nt) and second half (D2 2/2; 3333–5315 nt) of the 3′UTR were amplified by expand long range PCR and cloned between same sites. The GUAGU core consensus sequence was mutated to CAACA in the three putative binding sites at 2614 nt (M1), 3636 nt (M2), and 4881 nt (M3) of the wt chicken *D2* mRNA using overlap extension PCR.

### RNA EMSA

Histidine affinity-tagged mouse MSI1 protein was produced in Rosetta 2 *E. Coli* cells, purified on His-Pur Ni^2+^ affinity resin (Thermo Fisher Scientific), and dialyzed against 20 mM Tris/borate/EDTA (TBE) 5 mM DTT. Forty four base pairs-–long, 3′ biotin-labeled RNA oligonucleotide of *cDio2* 3′UTR mRNA was used in binding assay which includes two consensus binding site:

GUUUAUAGCUAGAUUAGUUUAGCUGAAAGUUAAGUAGUUCGCAA-[Btn] (Sigma-Aldrich). Binding reaction was performed under RNAse free conditions for 20 min at RT with LightShift Chemiluminescent RNA EMSA Kit (Thermo Fisher Scientific) REMSA 1× binding buffer, 0.125 mg/ml BSA, 80 fmol RNA probe, and 1.5 μg protein. To assess binding specificity, unlabeled RNA probe was added to binding reaction in 50× (4pmol) 200×(16pmol) 1250× (100pmol) molar excess. Five microliters of 20 μl binding reaction was loaded on native 6% polyacrylamide gel and was separated in 0.5× TBE (pH: 7.5), on 100 V, at 4 °C for 1 h. Semidry blot to nitrocellulose membrane (Immobilon Ny+) was run in 0.5× TBE overnight with 110 mA at 4 °C. Detection was carried out with LightShift Chemiluminescent RNA EMSA Kit (Thermo Fisher Scientific) according to kit protocol and captured on a KODAK Gel Logic 2200 Imaging System.

### Generation and treatments of primary astrocyte cell cultures

P1-2 pups of C57BL6 control and BP-MSI1-KO mice were sacrificed for cortical primary astrocyte isolation. Astrocytes were isolated according to previous protocol (59), seeded on poly-L-lysine coated (Sigma, P5899, 0.005 mg/ml final concentration) dishes, and maintained in standard astrocyte culture medium. After 7 days culturing, cells were seeded in 6-well plate in 4∗10^5^ cell density and were grown in normal astrocyte medium for 24 h. Cells were harvested and then stored as pellet on −80 °C until D2 activity measurements and gene expression analysis. For proliferation rate measurements and immunohistochemical staining of MSI1 protein, cells were seeded in 5∗10^4^ density to 4-well Lab-tek II chamber slides (Merk-Millipore) and 1 day later fixed with 4% PFA to determine the amount of MSI-1 protein in control and BP-MSI1-KO astrocytes with immunohistochemistry. To determine proliferation rate, control and BP-MSI1-KO astrocytes were treated with 10 μM BrdU for 24 h. To investigate the effect of T3 on proliferation rate, control astrocytes were grown in hormone free medium for 24 h and then were treated with 100 nM T3/vehicle (40 mM NaOH) and 10 μM BrdU for 24 h and fixed with 4% PFA before double labeling immunohistochemistry.

### Double labeling immunohistochemistry of GFAP and BrdU in primary astrocyte cultures and determination of proliferation rate

To detect proliferation rate of primary astrocytes under different conditions, astrocytes were double labeled for GFAP and BrdU. Four percent PFA fixed cells were permeabilized according to conventional techniques, followed by 20 min treatment in 2 M HCl. Mouse anti-GFAP antibody (Chemicon, MAB360, for validation see (60)) was used in 1:2000 dilution and sheep anti-BrdU serum (Abcam, ab1893) was used in 1:500 dilution. Immunoreactions were visualized by donkey anti-mouse Alexa 488 IgG and donkey anti-sheep Alexa 555 IgG (Invitrogen, 1:250 and 1:500, respectively). Proliferation rate was determined in four independent samples (chamber wells) per condition. GFAP-positive cells and GFAP/BrdU double positive cells were counted on four randomly selected, nonconfluent areas of chambers.

### D2 activity measurement

D2 activity was measured by counting deiodination generated I^125^ in sonicates of cell pellets and tissue samples, as described (36, 43, 61), in the presence of ∼100,000 cpm radiolabeled (I^125^) thyroxin, 1 nM or 0.1 nM cold T4 (CTX/MBH, respectively), 100 nM T3, 20 mM DTT, and 1 mM 6-n-propylthiouracil. Cell/tissue-specific conditions were as follows: H4 cells, 10 μg protein, 300 fmol T4, 300 min; MBH: 1 piece, 30 fmol T4, 360 min; CTX: 100 μg protein, 300 fmol T4, 240 min.

### Serum-free T4 and free T3 measurement

Whole blood was collected after decapitation and stored at 4 °C. Serum was aspirated after 15 min centrifugation at 3000 rcf at 4 °C and stored at −80 °C until fT4, fT3 measurement. fT4 and fT3 levels were measured with chemiluminescent-based enzyme immunoassay: AccuLite CLIA kit (Monobind Inc). Twenty microliters of each serum sample was measured in duplicate. All steps are prepared according to kit protocol, detecting chemiluminescence signal with Luminoscan Ascent Luminometer (Thermo Fisher Scientific) as described (36).

### Statistics

GraphPad Prism 9.2.0 software (https://www.graphpad.com/) was used to analyze data. Student’s two sample two-sided *t* test was used to analyze two groups with a 95% level of confidence.

## Data availability

All data are contained within the manuscript.

## Supporting information

This article contains supporting information.

## Conflict of interest

Antonio C. Bianco received consultant fees from AbbVie, Synthonics, Sention, Thyron, and Accella. The other authors declare that they have no conflicts of interests with the contents of this article.
