# Supplementary material for: The Musashi-1–type 2 deiodinase pathway regulates astrocyte proliferation
Source: J Biol Chem. 2024 Jun 13;300(7):107477. doi: 10.1016/j.jbc.2024.107477 (PMC11301063; doi:10.1016/j.jbc.2024.107477)
Supplement: Supplemental Figures [file mmc1.pdf]

Suppl. Figure 1.

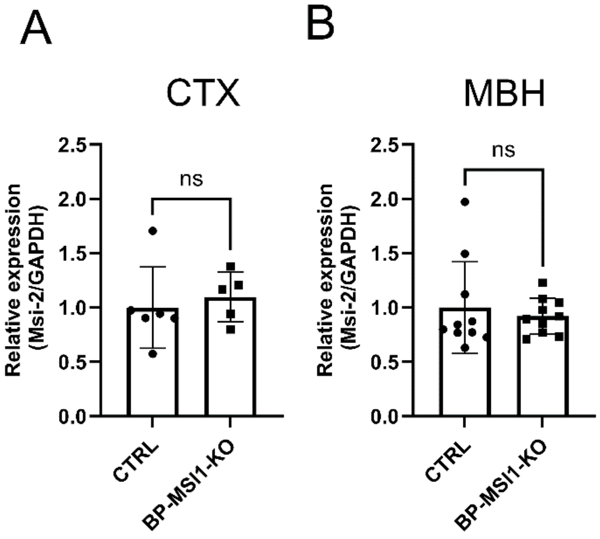

Msi2 expression is unchanged in the brain of BP-MSI1-KO mouse

Msi2 mRNA expression is unchanged in the cortex (CTX) and mediobasal hypothalamus (MBH) of the Brain Pituitary MSI1 -KO mouse (BP-MSI1-KO). Mean ± S.D., n≥5 in CTX, n=10 in MBH; Student’s t test
