# Supplementary material for: The Musashi-1–type 2 deiodinase pathway regulates astrocyte proliferation
Source: J Biol Chem. 2024 Jun 13;300(7):107477. doi: 10.1016/j.jbc.2024.107477 (PMC11301063; doi:10.1016/j.jbc.2024.107477)
Supplement: Supplemental Table [file mmc2.pdf]

Suppl. Table 1.

**Taqman Gene expression assay**	**Name**	**Assay ID**
Dio2	type 2 deiodinase	Mm00515664_m1
Msi1	musashi-1	Mm01203522_s1
Msi2	musashi-2	Mm01304232_s1
Tshβ	thyroid stimulating hormone, beta subunit	Mm03990915_g1
Hprt	hypoxanthine guanine phosphoribosyl transferase	Mm03024075_m1
Gapdh	glyceraldehyde-3-phosphate dehydrogenase	Mm99999915_g1

Data of used Taqman probes
